# Racial Disparities in Maternal Mortality Before, During, and After the COVID-19 Pandemic in the United States: A Difference-in-Difference Analysis

**DOI:** 10.7759/cureus.90416

**Published:** 2025-08-18

**Authors:** Kyosuke Kamijo, Misa Hayasaka, Tetsuya Kawakita

**Affiliations:** 1 Obstetrics and Gynecology, Nagano Prefectural Shinshu Medical Center, Nagano, JPN; 2 Obstetrics and Gynecology, Macon and Joan Brock Virginia Health Sciences at Old Dominion University, Norfolk, USA

**Keywords:** covid-19, health disparities, interrupted time series analysis, maternal mortality, pregnancy-related mortality, cross-sectional studies

## Abstract

Background

This study aimed to examine changes in racial disparities in pregnancy-related and maternal mortality before, during, and after the COVID-19 pandemic in the United States.

Methodology

This was a cross-sectional study using the Centers for Disease Control and Prevention Wide-ranging Online Data for Epidemiologic Research (CDC WONDER) data from January 2018 to June 2024. We focused on pregnancy-related and maternal mortality among Black and White individuals to examine racial disparities. The study periods were defined as pre-pandemic (January 2018-March 2020), pandemic (April 2020-March 2022), and post-pandemic (April 2022-June 2024). We applied interrupted time series analysis and difference-in-difference (DID) models to assess changes in mortality trends and disparities across three periods. DID estimates with 95% confidence intervals (CI) were reported.

Results

From January 2018 to June 2024, there were 3,694,282 Black and 17,284,929 White individuals who gave live birth. The pregnancy-related mortality ratio (PRMR) was 68.0 deaths per 100,000 live births among Black individuals (2,513 deaths) and 26.3 among White individuals (4,547 deaths). The maternal mortality ratio (MMR) was 46.5 for Black individuals (1,718 deaths) and 17.6 for White individuals (3,044 deaths). From the pre-pandemic to pandemic period, PRMR increased by 29.4 per 100,000 (95% CI = 19.8-39.1) among Black individuals and by 11.8 (95% CI = 8.2-15.4) among White individuals, with a DID of 17.6 (95% CI = 7.3-28.0). From the pre-pandemic to post-pandemic period, PRMR increased by 9.5 (95% CI = 3.2-15.9) for Black individuals and by 1.6 (95% CI = -0.4-3.7) for White individuals, with a DID of 7.9 (95% CI = 1.2-14.6). Results for MMR were consistent with those for PRMR.

Conclusions

These findings indicate a significant and sustained increase in PRMR and MMR among Black individuals, while rates among White individuals returned to near pre-pandemic levels. Racial disparities in pregnancy-related and maternal mortality widened during the COVID-19 pandemic and continued in the post-pandemic period.

## Introduction

The COVID-19 pandemic has significantly impacted maternal mortality, exacerbating pre-existing racial disparities [1]. Black women in the United States have historically experienced disproportionately higher maternal mortality rates compared to White women due to systemic inequities in healthcare access, quality of care, and social determinants of health [1]. The pandemic created additional challenges, including disruptions to routine prenatal care, healthcare system strain, and increased barriers to accessing care, raising concerns about further widening of the existing racial disparities [1].

Previous reports demonstrated the maternal mortality ratio (MMR) increased from 17.4 per 100,000 live births in 2018 to 32.9 in 2021, with Black individuals experiencing a rate of 69.9, over 2.5 times higher than the 26.6 observed among White individuals in 2021 [2]. While the overall rate decreased to 18.6 in 2023 after the pandemic [2], the pandemic may have further widened racial disparities in maternal mortality, with post-pandemic trends remaining unclear. This study aimed to examine changes in racial disparities in pregnancy-related and maternal mortality between Black and White individuals across the pre-pandemic, pandemic, and post-pandemic periods.

## Materials and methods

This cross-sectional study used publicly available data from the Centers for Disease Control and Prevention’s (CDC) Wide-ranging Online Data for Epidemiologic Research (WONDER) portal [3]. We obtained data on live births from the “Natality” datasets and mortality data from the “Underlying Cause of Death” records for the period of January 2018 to June 2024. The Natality datasets provided counts of live births occurring in the United States (50 states and the District of Columbia) to U.S. residents. The Underlying Cause of Death provided mortality data based on U.S. resident death certificates, listing a single underlying cause of death and demographic information. The analysis included individuals aged 15-44 years to avoid false positives associated with maternal mortality [4]. We focused our analysis on Black or African American (Black) and White populations to examine racial disparities because the database withholds monthly maternal death data when case counts fall below 10. Therefore, the mortality of other races was not consistently available for extraction.

The primary outcome was the pregnancy-related mortality ratio (PRMR), with MMR as a secondary outcome. We defined pregnancy-related death according to the CDC’s definition as “the death of a woman while pregnant or within 1 year of the end of pregnancy from any cause related to or aggravated by the pregnancy,” using International Classification of Diseases, Tenth Revision (ICD-10) codes corresponding to maternal deaths (ICD-10 codes: A34, O00-O99) to determine the numerator [5]. In addition, we defined maternal death according to the World Health Organization’s definition as “deaths from any cause related to or aggravated by pregnancy or its management (excluding accidental or incidental causes) during pregnancy and childbirth or within 42 days of termination of pregnancy, irrespective of the duration and site of the pregnancy,” using ICD-10 codes corresponding to maternal deaths (ICD-10 codes: A34, O00-O95, O98, O99) for the numerator [6]. The PRMR and MMR were calculated as the number of maternal deaths per 100,000 live births. Institutional Review Board review was deemed unnecessary, as only publicly available de-identified data were used.

Statistical methods

We evaluated outcomes by race across three periods: pre-pandemic (January 2018-March 2020) [1], pandemic (April 2020-March 2022), and post-pandemic (April 2022-June 2024) [7]. Using multivariable negative binomial regression with interaction terms between three periods and races, we assessed racial differences in the pandemic impact on mortality outcomes. We conducted an interrupted time series analysis (ITSA) to examine racial differences in trends across the three periods. We used ITSA to estimate the effects of the pandemic, adjusting for stable confounders. We also applied difference-in-difference (DID) to assess PRMR and MMR trends between Black and White populations [8]. To adjust for seasonality and temporal fluctuations, we incorporated Fourier terms into the regression models to capture monthly variations. We found that there was no significant difference in trends, which justifies only examining three periods to do DID analysis. To validate the key assumption of the DID model, we formally tested for parallel trends in the pre-pandemic period (January 2018-March 2020) between Black and White individuals. The analysis confirmed that there were no statistically significant differences in the pre-intervention mortality trends between the two groups, supporting the validity of our DID approach. DID estimates with 95% confidence intervals (95% CIs) were calculated, with the pre-pandemic period as a reference. Analyses were performed using Stata/IC 16.0 (StataCorp, College Station, TX, USA).

## Results

**Figure 1:**
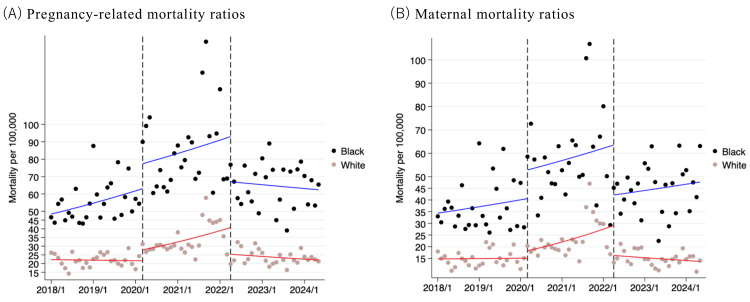
Charts showing monthly changes in pregnancy-related mortality ratios and maternal mortality ratios among Black and White individuals (January 2018 to June 2024). Dashed lines repre­sent pre-pandemic (January 2018 to March 2020), pandemic (April 2020 to March 2022), and post-pandemic (April 2022 to June 2024). Each line represents the predicted incidence of pregnancy-related mortality ratios (A) and maternal mortality ratios (B) (blue represents the results for Black individuals and red represents White individuals).

From January 2018 to June 2024, there were 3,694,282 Black individuals and 17,284,929 White individuals who gave live birth. The PRMR was 68.0 deaths per 100,000 live births among Black individuals (2,513 deaths) and 26.3 among White individuals (4,547 deaths). The MMR was 46.5 for Black individuals (1,718 deaths) and 17.6 for White individuals (3,044 deaths). Figure 1 illustrates the monthly changes in PRMRs and MMRs for Black and White individuals, showing similar trends between Black and White individuals in the three periods.

**Figure 2:**
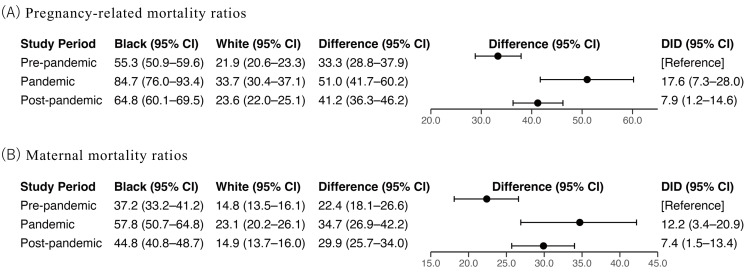
Pregnancy-related mortality ratios and maternal mortality ratios according to races and study period. CI: confidence interval; DID: difference-in-difference

Figure 2A presents PRMRs by race across the pre-pandemic, pandemic, and post-pandemic periods. From the pre-pandemic to the pandemic period, PRMR increased by 29.4 (95% CI = 19.8-39.1) for Black individuals and by 11.8 (95% CI = 8.2-15.4) for White individuals. The DID estimate of 17.6 (95% CI = 7.3-28.0) indicates a significantly larger increase among Black individuals. From the pre-pandemic to the post-pandemic period, PRMR increased by 9.5 (95% CI = 3.2-15.9) for Black individuals and by 1.6 (95% CI = -0.4-3.7) for White individuals, resulting in DID of 7.9 (95% CI = 1.2-14.6). Results of MMRs were consistent with the PRMR (Figure 2B).

## Discussion

This study found that racial disparities in both PRMR and MMR widened during the COVID-19 pandemic and persisted into the post-pandemic period. Even as overall mortality rates declined from their pandemic peak, the Black-White gap did not close, suggesting that the underlying structural inequities driving racial disparities in maternal health were not resolved by the end of the pandemic’s acute phase.

Our findings confirm that the pandemic amplified pre-existing structural inequities, which continue to drive maternal mortality disparities. While previous studies have documented the pandemic’s disproportionate impact on Black individuals [1,9], our analysis of a broader timeframe reveals these disparities were not a transient crisis but are deeply rooted in systemic issues. These include unequal access to high-quality prenatal and postpartum care, inconsistent health insurance coverage, and racially biased clinical practices [10-14]. The divergence in outcomes, where mortality rates for White individuals returned to pre-pandemic levels while those for Black individuals remained elevated, emphasizes how these systemic barriers uniquely harm marginalized populations even as a public health crisis subsides. Furthermore, these structural factors often intersect with clinical risks. Modifiable risk factors such as hypertension, obesity, and diabetes, conditions that increase the risk of maternal complications, are more prevalent among Black communities, partly due to the same systemic inequities in nutrition, environment, and healthcare access [15,16]. Therefore, effective policy interventions must be multifaceted, not only dismantling structural barriers but also addressing the underlying disparities in chronic health conditions through targeted public health initiatives and equitable clinical care [11-13,15,17,18].

A key finding of this study is the stark divergence in post-pandemic mortality trajectories between Black and White individuals. Although both Black and White individuals experienced increased PRMR during the pandemic, their trajectories diverged in the post-pandemic period. White individuals saw a return to pre-pandemic mortality levels, while mortality among Black individuals remained elevated. As a result, the racial disparity not only persisted but also widened further compared to pre-pandemic levels. To our knowledge, this is the first study to epidemiologically quantify the persistent post-pandemic gap using ITSA and DID modeling. These findings provide important evidence that the inequities in maternal health are not transient but rooted in deeper structural and systemic factors [13].

The sustained increase in mortality among Black individuals may reflect a toxic combination of post-pandemic factors. Black mothers may have faced persistent economic instability, reduced access to postpartum follow-up care as healthcare systems struggled to recover, and the cumulative psychological stress from both the pandemic and ongoing systemic racism, compounded by initial disparities in COVID-19 infection severity and healthcare disruptions, all of which exacerbate underlying clinical risks [19,20].

Our findings are particularly alarming when placed in an international context. Although other high-income countries document higher mortality among minority populations [21,22], the scale of the U.S. rates is unparalleled. While the national maternal mortality ratio in these peer nations is typically 10 per 100,000 live births or lower, the rate for Black individuals in the United States alone exceeded 50 in 2023 [2]. This reveals a systemic crisis where the mortality risk for one racial group in the United States is dramatically higher than the overall national risk in any comparable country.

This study has several limitations. First, we relied on CDC WONDER data, where pregnancy-related deaths are identified using the pregnancy checkbox on the U.S. standard death certificate. This method is subject to coding errors and misclassification. To address this limitation, we restricted our study population to women aged 15 to 44 years and the study period to 2018 and after, when the implementation of the revised certificate was completed and a new coding method was adopted. However, residual coding errors may still be present [23]. Second, CDC WONDER does not provide individual-level data, which limited our ability to perform multivariate analyses. This prevented us from adjusting for known individual-level confounders such as education, income, insurance status, and clinical comorbidities (e.g., hypertension, diabetes), which are known to influence maternal outcomes and differ between racial groups [15,16]. Third, due to data suppression for small counts, our analysis was limited to Black and White individuals, preventing a broader understanding of disparities across other racial and ethnic groups. Fourth, the use of monthly aggregated data may have smoothed over more acute fluctuations in mortality linked to specific events, such as peaks in COVID-19 transmission or abrupt policy changes. Fifth, our outcome definitions excluded pregnancy-associated deaths not coded as pregnancy-related, such as those from suicide, overdose, or homicide, which have become increasingly important contributors to maternal mortality [24-26]. Finally, because COVID-19 is recorded as a contributory cause of death in CDC WONDER data, we were unable to determine whether the maternal deaths were directly caused by COVID-19 infection. Despite these limitations, this study provides a foundation for further investigation using more comprehensive, multilevel data to clarify the drivers of racial disparities and inform targeted interventions.

## Conclusions

The Black-White disparity in maternal mortality widened during the COVID-19 pandemic and, critically, has persisted in its aftermath. Our analysis shows that while mortality rates for White individuals returned to pre-pandemic levels, rates for Black individuals remained significantly elevated. This demonstrates that the pandemic did not merely exacerbate existing inequities but entrenched them, creating a lasting legacy of harm that extends beyond the acute public health emergency. The divergent recovery paths highlight an urgent need for comprehensive policy interventions. Such interventions must move beyond temporary fixes to address the root causes of disparity, tackling both the systemic inequities that create barriers to care and the clinical risk factors that disproportionately affect Black communities.
